# Nitric oxide promotes energy metabolism and protects mitochondrial DNA in peaches during cold storage

**DOI:** 10.3389/fpls.2022.970303

**Published:** 2022-10-06

**Authors:** Yuanyuan Ren, Shuhua Zhu

**Affiliations:** College of Chemistry and Material Science, Shandong Agricultural University, Taian, China

**Keywords:** nitric oxide, mitochondria, energy metabolism, mitochondrial DNA, peach, cold storage

## Abstract

The mitochondria are important organelles related to energy metabolism and are susceptible to oxidative damage. In this experiment, peaches (*Prunus persica*) were treated with distilled water (as the control), 15 μmol L^−1^ of nitric oxide (NO), and 20 μmol L^−1^ of carboxy-PTIO (NO scavenger). The changes in mitochondrial physiological indicators, energy metabolism process, and mitochondrial DNA (mtDNA) damage and repair were quantified. Compared with the control, NO treatment reduced mitochondrial oxygen consumption and the reactive oxygen species content, increased mitochondrial respiration control rate, and promoted energy metabolism by influencing the activities of citrate synthase, aconitase, isocitrate dehydrogenase, and α‐ketoglutarate dehydrogenase in the tricarboxylic acid cycle and ATPase activity in peach mitochondria. NO treatment also maintained the relative copy number of mtDNA and the relative amplification of long PCR in peaches, decreased the level of 8-hydroxy-2 deoxyguanosine, and upregulated the expression of *PpOGG1*, *PpAPE1*, and *PpLIG1*. These results indicated that exogenous NO treatment (15 μmol L^−1^) could reduce mtDNA oxidative damage, maintain mtDNA molecular integrity, and inhibit mtDNA copy number reduction by reducing the reactive oxygen species content, thereby promoting mitochondrial energy metabolism and prolonging the storage life of peaches at low temperatures.

## Introduction

As important semi-autonomous organelles, the mitochondria are integral to numerous metabolic pathways and play essential roles in plants (Liberatore et al., 2016). The most basic function of the mitochondria is to carry out energy metabolism (Bratic and Trifunovic, 2010). The tricarboxylic acid (TCA) cycle is one of the most important cycles in the mitochondria: it is the only way for carbohydrates, lipids, and amino acids to carry out the final chemical reaction in cells and the “meeting point” for the chemical reactions of these three nutrients in cells (Lu et al., 2018). It can also provide small molecule precursors for other metabolisms, such as amino acid and sugar synthesis (Lu et al., 2018). ATPase powers ATP synthesis and is responsible for the reversible catalysis of ADP and Pi to ATP (Dautant et al., 2018).

Mitochondrial DNA (mtDNA) is a genome that exists in the mitochondria, is independent of the extrachromosomal nucleus, and is capable of self-replication, transcription, and coding (Roger et al., 2017). A mitochondrion contains multiple copies of mtDNA, and the copy numbers of mtDNA can change depending on the energy requirements of the cells (Barazzoni et al., 2000), the developmental stage (Niazi et al., 2019), and the environmental stress (Ahmad et al., 2010; Zhao et al., 2018). Environmental stresses can cause reactive oxygen species (ROS) to burst, which will lead to oxidative damage (Li et al., 2018). As naked DNA lacks protein protection, mtDNA is highly susceptible to damage by surrounding ROS due to its location near the electron transport chain (ETC) (Minibayeva et al., 2012). ROS induces oxidative base lesions and the degradation of mtDNA, causing mtDNA mutations (Shokolenko et al., 2009). Also, mtDNA damage directly causes aging that results in increased ROS, causing autophagy and cell death (Van Houten et al., 2016; Baumann, 2019). Excessive ROS results in a massive accumulation of 8-hydroxy-2′-deoxyguanosine (8-OHdG), considered the most sensitive symbolic product of DNA oxidative damage (Richter, 1995; Cioffi et al., 2019). Oxidative damage to the mtDNA also leads to mitochondrial oxidative phosphorylation dysfunction, impaired cellular energy metabolism, and altered mitochondrial integrity, which may trigger apoptosis (Jiao et al., 2014).

DNA damage repair is a complex and delicate regulatory mechanism in the organism. Base excision repair (BER) is the major repair pathway in the plant responsible for eliminating spontaneous hydrolytic, alkylation, deamination, and DNA oxidative damage, thereby sustaining genomic integrity (Jeppesen et al., 2011; Kim and Wilson, 2012; Morales Ruiz et al., 2018). When the damaged or modified bases occur in the DNA strands, the N-glycosidic bond will be cleaved by damage-specific DNA glycosylases, then an apurinic/apyrimidinic (AP) site is generated, finally repaired by various enzymes. The complete BER pathway has been demonstrated in the mitochondria of potato tubers responding to oxidative stress, and 8-oxoG-DNA glycosylase (OGG1), an apurinic/apyrimidinic (AP) endonuclease 1 (APE1), and DNA ligase I (LIG1) are found to participate in the BER pathway (Ferrando et al., 2019).

As a bioactive molecule, nitric oxide (NO) affects ATP synthesis in the respiratory chain, mediates free radicals, inhibits mitochondrial respiration, and regulates many mitochondrial functions (Poderoso et al., 2019). NO is also a biologically active ROS scavenger that prevents plants from suffering severe oxidative threats (Hasanuzzaman et al., 2018). The mitochondria are not only the target of NO but also the source of NO, and NO can maintain mitochondrial integrity by reducing oxidative damage (Jing et al., 2016). NO activates the antioxidant system to defend against excessive ROS in plants (Sahay and Gupta, 2017). Moreover, NO, acting as the second messenger at opportune concentration, protects the mitochondria in different pathways (Litvinova et al., 2015). NO treatment has been shown to maintain the mitochondrial ETC and alleviate mitochondrial oxidative damage in peach fruit (Wang et al., 2021). This paper reported the regulation by NO on TCA, ATPase, and mtDNA in peach fruit.

## Materials and methods

### Plant material and isolation of the mitochondria

Peaches (*Prunus persica*) with similar size, no pests, and no mechanical damage were harvested from Xintai, Shandong, China. The reagent concentrations and duration of treatment were determined according to a previous experiment (Jing et al., 2016), peaches were soaked for 30 min in each treatment, and three treatments were performed: distilled water, 15 μmol L^−1^ NO solution, and 20 μmol L^−1^ c-PTIO (NO scavenger). The treated peaches stored at 0°C were sampled once a week, and the phenotypic appearances of peaches after treatment are shown in the **Supplementary Materials**. The mitochondria of peaches were extracted using the Mops-KOH buffer and quantized using Coomassie brilliant blue solution as described by Jing et al. (2016), and the purity and integrity of the mitochondria were determined according to Millar et al. (2001). The total protein concentration of purified mitochondria with integrity was adjusted to 100 μg ml^−1^ for the following experiments.

### Determination of mitochondrial oxygen consumption and mitochondrial respiratory control ratio

The mitochondrial oxygen consumption was measured as follows: after incubation for 2 min, the reaction medium (0.7 ml, pH 7.4, contained 0.4 mol L^−1^ mannitol, 0.2 mol L^−1^ sucrose, 10 mmol L^−1^ potassium chloride, 10 mmol L^−1^ magnesium chloride, and 10 mmol L^−1^ Tris–HCl) was mixed with the mitochondria (20 μg). Each dissolved oxygen content curve was recorded for 10 min in the Oxygraph Plus System (Hansatech, Britain). Oxygen consumption was obtained with the slope of the curve and was expressed as mmol O_2_ min^−1^ g^−1^ protein.

The oxygen consumption curve was continuously recorded after the above solution was mixed with 20 μl of reaction substrate (a mixture of 2.5 mmol L^−1^ of sodium malate and sodium pyruvate) and 5 μl of ADP (60 mmol L^−1^). The slope of the curve displayed the ADP respiration rate (state III). The mitochondrial respiration control rate (RCR) was expressed as the ratio of the ADP respiration rate (state III) to the ADP-depleted respiration rate (IV state).

### Determination of mitochondrial H_2_O_2_, ·OH, and O_2_^−^·contents

The H_2_O_2_ content was determined according to Zheng et al. (2009). The mitochondria (50 μg) were mixed with 0.5 ml of NH_4_OH and 0.5 ml of TiSO_4_ (5%, v/v). After being centrifuged at 12,000×*g* for 10 min, the precipitate was mixed with 1 ml of H_2_SO_4_ (2 mol L^−1^) at 415 nm using a spectrophotometer.

The ·OH content was measured as described by Giulivi et al. (1995). The mitochondria (50 μg) were mixed with 2 ml of deoxyribose (2.5 mmol L^−1^) and reacted at 37°C for 60 min. Next, the mixture was boiled for 30 min following the addition of acetic acid (0.5 ml) and 0.5 ml of 1% thiobarbituric acid and then immediately cooled on ice for 10 min. The ·OH content was measured in a spectrophotometer at 532 nm.

The O_2_^−^· content was tested as described by Zheng et al. (2009). The mitochondria (50 μg) were incubated with 0.5 ml of 10 mmol L^−1^ hydroxylamine hydrochloride solution for 30 min at 25°C. α-Naphthylamine (7 mmol L^−1^) and P-aminobenzene sulfonic acid (17 mmol L^−1^) were added for a further 30 min. The O_2_^−^· content was determined as the absorbance at 530 nm and expressed as mol g^−1^ protein.

### Determination of energy metabolism-related enzyme activities

Citrate synthase (CS) activity was determined as the absorbance change at 412 nm (Schmidtmann et al., 2014). The mitochondria (50 μg) were incubated with 0.2 mmol L^−1^ of acetyl-CoA (10 μl) in 0.1 mL of 1 mmol L^−1^ DTNB (in 100 mmol L^−1^ of Tris–HCl, pH 8.0), and the reaction was started after the addition of 0.2 mmol L^−1^ of oxaloacetate (0.1 ml). One unit of CS activity (U) was defined as the amount required to change the absorbance at 412 nm by 0.01 within 1 min.

Aconitase (ACN) activity was tested as described by Middaugh et al. (2005). The mitochondria (50 μg) were incubated at room temperature in a 2-ml reaction containing 100 mmol L^−1^ of Tris–HCl (pH 7.3), 1 mmol L^−1^ of DTT, 1 mmol L^−1^ of phenylmethylsulfonyl fluoride, 10 mmol L^−1^ of citrate, and 20 mmol L^−1^ of malonate. ACN activity was monitored by following the formation of cis-aconitate at 240 nm.

Isocitrate dehydrogenase (IDH) activity was determined as described by Jenner et al. (2001). The change rate of absorbance at 340 nm was monitored in the following mixture: mitochondrial suspension (50 μg), 0.5 ml of 50 mmol L^−1^ Tris–HCl [pH 7.6, contained 1.5 mmol L^−1^ of NAD, 6.3 mmol L^−1^ of MnCl_2_, and 0.05% (v/v) Triton X-100]. The reaction was started by the addition of 15 mmol L^−1^ of isocitrate (0.2 ml).

The measurement of α-ketoglutarate dehydrogenase (α-KGDHC) activity was based on Nulton Persson et al. (2003). After the mitochondria (0.5 ml) were mixed with 2 ml of 50 mmol L^−1^ Mops buffer (pH 8.0, contained 5 mmol L^−1^ of MgCl_2_, 40 μmol L^−1^ of rotenone, 2.5 mmol L^−1^ of α-ketoglutarate, 0.1 mmol L^−1^ of CoA, 0.2 mmol L^−1^ of thiamine pyrophosphate, 1 mmol L^−1^ of NAD^+^, and 0.1% Triton X-100), α-KGDHC activity was measured spectrophotometrically at the rate of NADH production at 340 nm. The activities of H^+^-ATPase and Ca^2+^-ATPase were measured referring to the method of Jin et al. (2014). The mitochondria (50 μg) were added to 2-ml reaction reagents (contained 50 mmol L^−1^ of potassium chloride and 3 mmol L^−1^ of magnesium sulfate or 10 mmol L^−1^ of magnesium chloride) and reacted in a water bath (30°C) for 30 min. The reaction was stopped by adding 0.1 ml of 50% TCA and 0.1 ml of 2.5% ammonium molybdate, and the activities of H^+^-ATPase and Ca^2+^-ATPase were expressed as the release of inorganic phosphate (Pi) resulting from the hydrolysis of ATP at 660 nm.

### Determination of the relative mtDNA copy number

The relative mtDNA copy number was characterized by examining the amplification of *PpNAD1* and *ACTIN via* qRT-PCR and calculated using the 2^−ΔΔCt^ method. The primer sequences used are shown in **Table 1**.

**Table 1:** DNA primers used for real-time PCR and long PCR.

Gene	Forward primer (5′ to 3′)	Reverse primer (5′ to 3′)	Product (bp)
*PpNaD1*	TTTAGTTGTGGGTCATAGGGC	TCGTCCATTCGTTGAGTGATC	184
*PpACTIN*	CTGGTATTGTGCTGGACTCTG	CCCTCTTTCGGTGAGAATCTTC	146
*Pplong*	AGAGGGCTCTGGTTCAAGC	CACTCTTCCTTGCGATGCCT	10,054
*Ppshort*	AGCAGGTTTGTGACGCTCTC	ACCACCTCACTCCAAAGAAAGA	250
*PpOGG1*	CACCACCACCTCCGAAAC	GCACCGCCCAAATAGC	138
*PpAPE1*	GGTGCAGTGCAGGACTCTC	TTGGAGCTACTGCAAAGCCT	97
*PpLIG1*	CTGCGGACTTGACTATTAGCC	AGGTTTATCTTCCCGAACACG	113
*PpTUB*	TGACAAGACCGTTGGTGGAG	GGAAGAGCTGGCGGTAAGTT	149

### Quantification of mtDNA damage

Long PCR was performed using an *ApexHF* HS DNA Polymerase CL Kit (Accurate Biotechnology, China) in a MyCycler PCR system (Bio-Rad, USA) for mtDNA damage evaluation, according to the protocol described previously (Zhou et al., 2011). The primers are listed in **Table 1**. The final long PCR cycling parameters followed the manufacturer’s recommendations: initial denaturation of 1 min at 94°C followed by 10 s at 98°C, 15 s at 60°C, and 12 min at 68°C for 28 cycles. The PCR cycling parameters for small fragments underwent the following profiles: initial denaturation of 1 min at 94°C followed by 10 s at 98°C, 15 s at 60°C, and 30 s at 68°C for 25 cycles. Each PCR product was quantified using ImageJ.

### Determination of the level of 8-OHdG in mitochondrial DNA

The level of 8-OHdG was measured using a double-antibody sandwich method according to the ELISA kit of 8-OHdG for the plant (MeiLianShengWu, Shanghai, China). Standard 8-OHdG was detected within the range of 1.2–40 pg ml^−1^, and the sample was diluted 10 times during the experiment. The absorbance was determined at 450 nm within 15 min after the termination fluid was added, and the result was expressed as μg g^−1^ protein.

### Determination of the expression of genes related to BER

**Table 1** lists the qRT-PCR primer sequences. Total RNA was extracted and then reverse-transcribed by an Evo M-MLV RT Kit (Accurate Biology, China) (Wang and Stegemann, 2010). The qPCRs were carried out using a SYBR Green Premix Pro Taq HS qPCR Kit (Accurate Biology, China). The expression levels were calculated with the 2^−ΔΔt^ formula (*PpTUB*: the reference gene).

### Statistical analysis

The data for statistical analysis were obtained from at least three independent experiments. Data were presented as means ± SD and processed by an analysis of variance (ANOVA), with *P <*0.05 indicating significant differences, based on the least significant difference (LSD) test.

## Results

### Changes in the mitochondria of peaches

**Figure 1:**
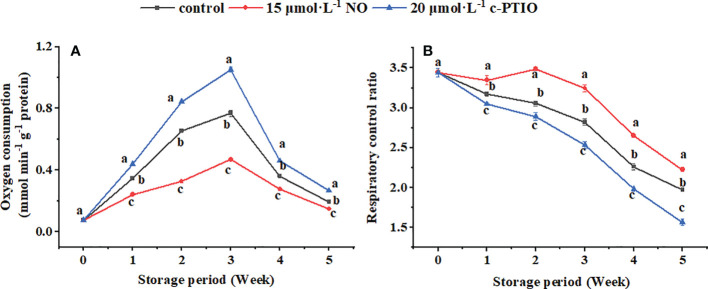
Changes in mitochondrial oxygen consumption **(A)** and mitochondrial respiratory control ratio **(B)** in peaches. Error bars indicate standard errors (*n* = 3). Different letters indicate significant differences among different treatments (*P* < 0.05).

The mitochondrial respiratory oxygen consumption of peaches peaked at week 3 and then declined during storage (**Figure 1A**). NO decreased but c-PTIO increased the mitochondrial oxygen consumption of peaches (*P* < 0.05). At week 5, the mitochondrial oxygen consumption of the NO-treated peach fruit was 75.74% compared with that of the control and 55.02% compared with that of the c-PTIO-treated fruit. The mitochondrial RCR decreased in the first week and remained relatively stable during storage in each treatment (**Figure 1B**). The mitochondrial RCR in the NO treatment was 1.15 times higher than that in the control at week 3. c-PTIO decreased the mitochondrial RCR of peaches (*P* < 0.05), and especially at weeks 4 and 5, the mitochondrial RCR was only 74.81% and 70.42%, respectively, compared with that of the control.

### Change in the mitochondrial H_2_O_2_, ·OH, and O_2_^−^· content

**Figure 2:**
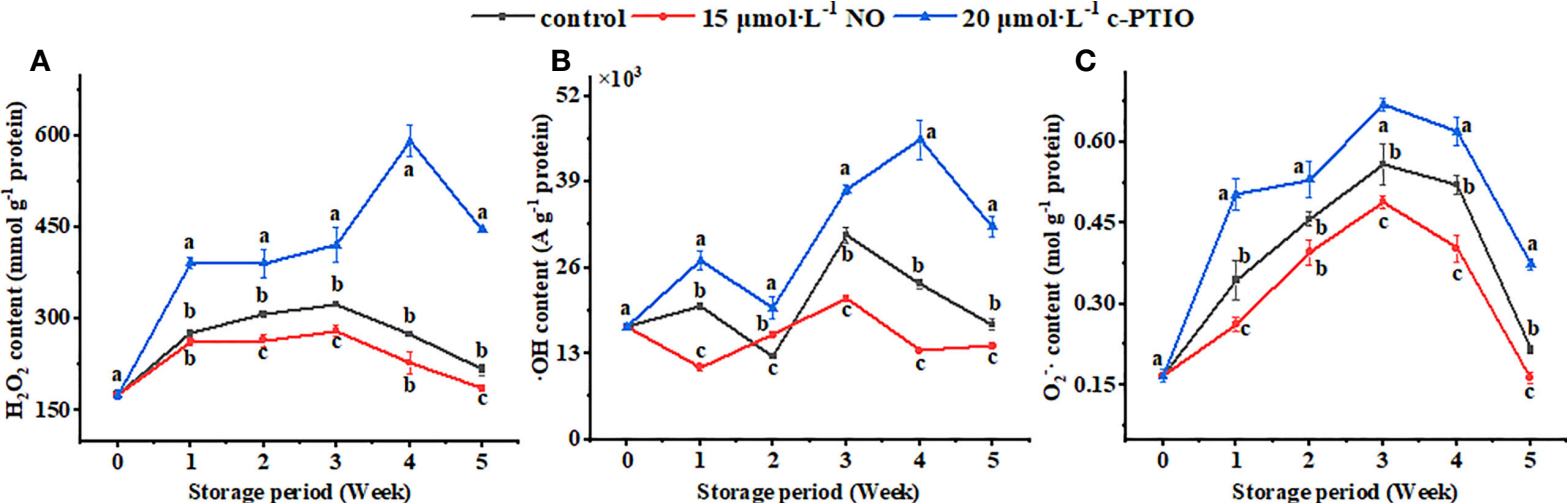
Changes in H_2_O_2_ **(A)**, ·OH **(B)**, and O_2_^−^· **(C)** contents in peach mitochondria. Error bars indicate standard errors (*n* = 3). Different letters indicate significant differences among different treatments (*P* < 0.05).

The H_2_O_2_ content in the mitochondria first increased and then decreased, and the c-PTIO treatment obviously (*P* < 0.05) increased the H_2_O_2_ content (**Figure 2A**). At week 4, the H_2_O_2_ content in the c-PTIO-treated mitochondria was 2.49 times higher than that in the NO-treated mitochondria.

The maximum ·OH content appeared at week 3 in the control and NO treatment, and the maximum appeared at week 4 in the c-PTIO treatment (**Figure 2B**). At week 3, the ·OH content in the NO treatment was 69.13% compared with that in the control.

The maximum O_2_^−^· content appeared in week 3 (**Figure 2C**). NO treatment inhibited the increase in the O_2_^−^· content (*P* < 0.05). In the NO treatment, the O_2_^−^· content was 73.15% compared with that in the c-PTIO treatment at week 3.

### Change in energy metabolism-related enzyme activities

**Figure 3:**
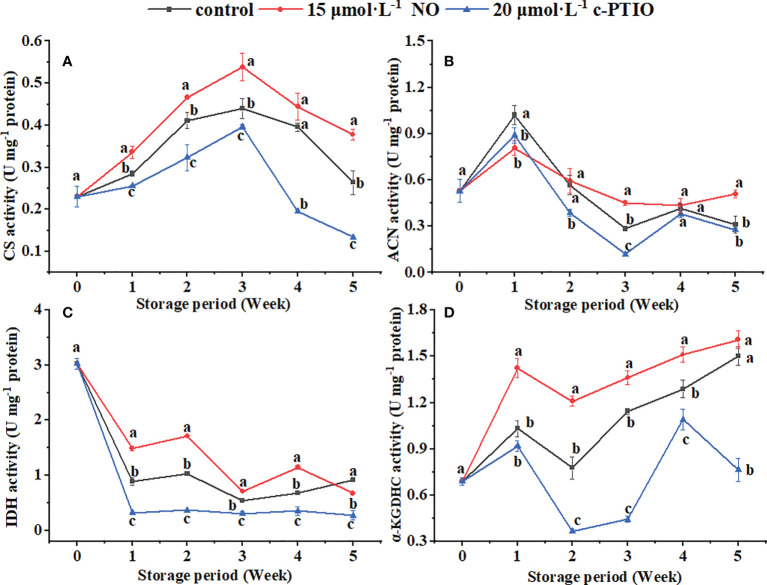
Changes in the CS **(A)**, ACN **(B)**, IDH **(C)**, and α-KGDHC **(D)** activities in peach mitochondria. Error bars indicate standard errors (*n* = 3). Different letters indicate significant differences among different treatments (*P* < 0.05).

The CS activity peaked at week 3 (**Figure 3A**). NO treatment increased the CS activity except at week 4 (*P* < 0.05). At week 5, the CS activity in the NO treatment was 2.82 times higher than that in the c-PTIO treatment.

The ACN activity in the control was the highest at week 1 (**Figure 3B**). In the NO treatment, the ACN activity was higher than in the control at weeks 3 and 5 (*P* < 0.05). At week 3, the ACN activity in the NO-treated peach mitochondria was 3.76 times higher than that in the c-PTIO-treated peach mitochondria.

The IDH activity decreased rapidly at week 1 and changed less after that (**Figure 3C**). Except for week 5, NO treatment increased the IDH activity (*P* < 0.05). The IDH activity in the NO treatment was 4.62 times higher than that in the c-PTIO treatment at week 2.

The α-KGDHC activity kept increasing during the storage period in the NO treatment and control except at week 2 (**Figure 3D**). In the c-PTIO treatment, the trend in α-KGDHC activity changes was consistent with the other two treatments during the first 4 weeks, but the activity began to decline at week 5. The α-KGDHC activity in the NO treatment was 1.55 times higher than that in the control at week 2.

**Figure 4:**
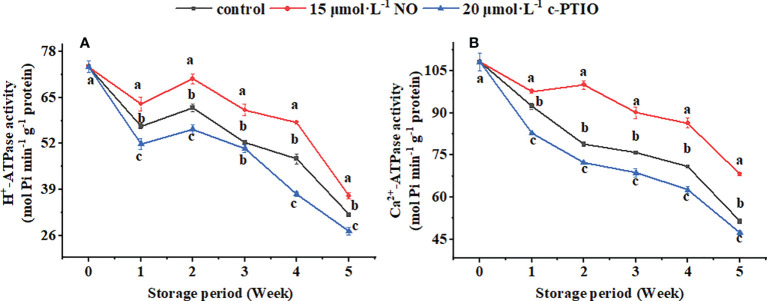
Changes in the H^+^-ATPase **(A)** and Ca^2+^-ATPase **(B)** activities in peach mitochondria. Error bars indicate standard errors (*n* = 3). Different letters indicate significant differences among different treatments (*P* < 0.05).

The H^+^-ATPase activity showed a downward trend during the storage period except for weeks 1 to 2 (**Figure 4A**); c-PTIO treatment inhibited its activity (except week 3) (*P* < 0.05). Especially at week 4, the H^+^-ATPase in the c-PTIO treatment was only 73.16% compared with that in the NO treatment.

The Ca^2+^-ATPase activity remained generally decreased except for the NO treatment at weeks 1 to 2 (**Figure 4B**). NO treatment maintained the Ca^2+^-ATPase activity (*P* < 0.05). The Ca^2+^-ATPase activity in the control and c-PTIO treatment was 78.87% and 72.25% compared with that in the NO treatment at week 2, respectively.

### Change in the mtDNA of peaches

**Figure 5:**
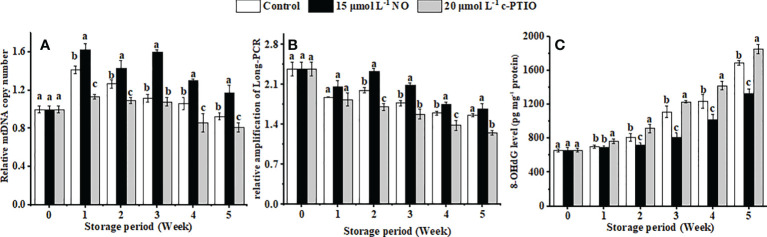
Changes in the mtDNA copy number **(A)**, relative amplification of long PCR **(B)**, and the level of 8-OHdG in mtDNA **(C)** in peaches. Error bars indicate standard errors (*n* = 3). Different letters indicate significant differences among different treatments (*P* < 0.05).

The relative mtDNA copy number of the peaches firstly increased and then declined during storage (**Figure 5A**). NO postponed the changes of the relative mtDNA copy number, and at week 5, it was 1.44 times higher than that in the c-PTIO treatment (*P* < 0.05).

mtDNA damage gradually increased with storage time, and NO treatment suppressed the aggravation of the damage (**Figure 5B**) (*P* < 0.05). The relative amplification of long PCR in the NO treatment was 1.36 times higher than that in the c-PTIO treatment at week 2.

8-OHdG in the mtDNA in peaches increased, and NO could dramatically inhibit the growth in the level of 8-OHdG in mtDNA (**Figure 5C**) (*P* < 0.05). Especially at week 5, the level of 8-OHdG in the NO-treated peaches was 78.78% compared with that in the control, and in the c-PTIO treatment, it was 1.10 times higher than that in the control.

### Changes in the expression of genes related to BER

**Figure 6:**
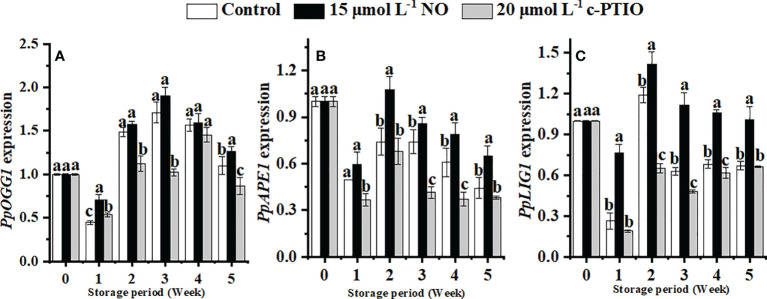
Changes in the expression levels of *PpOGG1* **(A)**, *PpAPE*1 **(B)**, and *PpLIG1* **(C)** in peaches. Error bars indicate standard errors (*n* = 3). Different letters indicate significant differences among different treatments (*P* < 0.05).

The expression of *PpOGG1* peaked at week 3 in peaches (**Figure 6A**). NO treatment upregulated the expression of *PpOGG1* (*P* < 0.05). The peak value of NO-treated peaches was 1.17 times higher than that of the control.

The expression of *PpAPE1* was higher in the NO treatment than in others (**Figure 6B**). At week 2, the expression of *PpAPE1* in NO-treated peaches was 1.52 times higher than that in the control.

NO treatment significantly (*P* < 0.05) maintained the expression of *PpLIG1* (**Figure 6C**). In the NO treatment, the expression of *PpLIG1* was 2.18 times higher than that in the c-PTIO treatment at week 2.

## Discussion

The mitochondria of peaches treated with NO had the lowest oxygen consumption compared with the other two treatments. However, the RCR was the highest (**Figure 1**). During storage, the mitochondria are continuously energized and continuously form ROS. The accumulation of ROS causes damage to the mitochondria, which leads to mitochondrial apoptosis (Gu et al., 2016). mtDNA damage is due to ROS accumulation in the organelles, so reducing ROS can reduce mitochondrial oxidative damage and maintain cell stability (Shaughnessy et al., 2014). Exogenous NO reduced the ROS content (**Figure 2**). Similar results were also found in wheat (Si et al., 2017), Hami melon (Zhang et al., 2017), cornelian cherry fruit (Rabiei et al., 2019), and peaches (Huang et al., 2019a). In summary, NO treatment maintained the quality of the mitochondria and reduced the ROS content.

The TCA cycle is integral to harvesting energy (Shen et al., 2019), and its rate is thought to be determined by CS, IDH, and α-KGDHC (Krebs, 1970; Mastrogiacomo et al., 1996; Huang et al., 2019b). NO treatment has been shown to relieve seed aging and promote seed germination by increasing CS activity (Mao et al., 2018). Moreover, NO treatment alleviates salt stress by increasing the CS and IDH activities in *Brassica napus* L. (Zhang et al., 2021). α-KGDHC is not only the rate-limiting enzyme in the TCA cycle but also a target of ROS, and NO can increase its activity by S-nitrosylation (Sun et al., 2007). CS, IDH, and α-KGDHC were also increased in peach mitochondria treated with NO in this study, and c-PTIO treatment showed opposite results (**Figures 3A–D**). NO is generally considered an inhibitor of ACN, but with further research, NO is shown to reversibly inactivate ACN by controlling the loss of Fe–S clusters (Navarre et al., 2000). NO at a higher concentration than the physiological concentration is the real reason for the inactivation of ACN. At the same time, the reaction time and substrate concentration can affect NO regulation on the ACN activity (Tórtora et al., 2007). In this study, NO treatment showed inhibition first and then promotion of ACN, which may be due to the combined effect of time and NO concentration on ACN (**Figure 3B**). H^+^-ATPase hydrolyzes ATP to generate ADP and free phosphate ions to release energy while establishing a transmembrane electrochemical gradient and transmembrane proton driving force. At the same time, ATP is synthesized under the catalysis of the transmembrane proton electrochemical potential (Olsen et al., 2009; Esparza and Cuezva, 2018). Exogenous NO has long been shown to enhance the salt tolerance of wheat seedlings by increasing the activity of H^+^-ATPase (Fan et al., 2013). In this study, the H^+^-ATPase activity was significantly increased in the NO treatment (**Figure 4A**). These data suggested that NO treatment might increase the level of energy release and proton electrochemical gradient by increasing the activity of H^+^-ATPase and increase the synthesis of ATP through a sufficient proton electrochemical gradient. Ca^2+^-ATPase is related to cell homeostasis: it can use the energy generated by ATP hydrolysis to regulate the concentration of Ca^2+^, avoid excessive accumulation of Ca^2+^, and then damage the mitochondria, thereby limiting energy synthesis (Anil et al., 2008; Jin et al., 2014). The Ca^2+^-ATPase activity was increased in the NO-treated peach mitochondria (**Figure 4B**). This suggested that NO treatment might maintain ion homeostasis by increasing the activity of Ca^2+^-ATPase, thereby maintaining mitochondrial function and maintaining energy metabolism and energy supply. The above result revealed that NO treatment could promote energy metabolism by increasing the rate-limiting enzyme activity in the TCA cycle and ATPase activity.

The mtDNA copy number can be maintained by mitochondrial gene expression and ATP (Cadenas, 2018). A large amount of ROS could be produced when peaches are stored in a low-temperature condition, which could damage the mitochondrial integrity of peaches, and the damaged mitochondria would rerelease ROS, exacerbating the damage to other mitochondria in a vicious circle. mtDNA in the c-PTIO treatment was highly damaged, with less relative mtDNA copy number and relative amplification of long PCR, than NO (**Figures 5A, B**). It was consistent with the fact that the increase in the content of organelle-generated ROS could lead to a decrease in the retention or the degradation of mtDNA. 8-OHdG, as a marker of mtDNA damage, is formed by the interaction of HO• with the guanine of the DNA strand (Valavanidis et al., 2009). The level of 8-OHdG is positively correlated with ROS, even the degree of DNA oxidative damage (Fraga et al., 1990; Kondo et al., 2000). NO decreased the level of 8-OHdG in mtDNA, so NO might alleviate the oxidative damage of mtDNA in peaches (**Figure 5C**). The above results showed that NO treatment could protect the mtDNA.

Although the idiographic DNA repair mechanisms are still poorly understood in plant organelles, the BER pathway is deemed to remove oxidative damage in plant mitochondria, such as *Arabidopsis* (Roldán Arjona et al., 2019), *Solanum tuberosum* tubers (Ferrando et al., 2019), and *Zea mays* (Kumar et al., 2014). In this research, NO upregulated the expression of *PpOGG1*, *PpAPE1*, and *PpLIG*1 effectively compared with the other treatments (**Figure 6**). The upregulation of OGG1 coincides with oxidative DNA damage with a lower background mutation (Wu et al., 2004; Macovei et al., 2011), and the overexpression of APE1 markedly enhances BER for maintaining DNA integrity (Dobson et al., 2000). Experimental evidence indicates that LIG1 is the only ligase that can have nick-closing functions for plant BER (Foyer and Noctor, 2003), and the activity of LIG1 increased after mtDNA suffering from oxidative damage (Ferrando et al., 2019). NO was better able to repair DNA oxidative damage by increasing the expression levels of *PpOGG1*, *PpAPE1*, and *PpLIG1* and further enhanced the protein expression of BER. These results proved that NO does protect DNA from oxidative damage and maintain DNA integrity.

## Conclusion

NO reduced mitochondrial oxygen consumption and ROS content, increased mitochondrial RCR, and promoted energy metabolism by influencing CS, ACN, IDH, and α-KGDHC activities in the TCA cycle and ATPase activity in peach mitochondria. NO also maintained the relative copy number of mtDNA and the relative amplification of long PCR in peaches, decreased the level of 8-OHdG, and upregulated the expression of *PpOGG1*, *PpAPE1*, and *PpLIG1*. These results indicated that exogenous NO treatment (15 μmol L^−1^) could reduce mtDNA oxidative damage, maintain molecular integrity, and inhibit mtDNA copy number reduction by reducing the ROS content, thereby promoting mitochondrial energy metabolism and prolonging the storage life of peaches at low temperatures.

## Data availability statement

The original contributions presented in the study are included in the article/**Supplementary Material**, further inquiries can be directed to the corresponding author.

## Author contributions

YR: Investigation, Writing - original draft, Formal analysis. SZ: Funding acquisition, Conceptualization, Supervision, Resources, Writing - review and editing. All authors contributed to the article and approved the submitted version.

## Funding

The authors thank the National Natural Science Foundation of China for the funding (31770724, 32071808).

## Conflict of interest

The authors declare that the research was conducted in the absence of any commercial or financial relationships that could be construed as a potential conflict of interest.

## Publisher’s note

All claims expressed in this article are solely those of the authors and do not necessarily represent those of their affiliated organizations, or those of the publisher, the editors and the reviewers. Any product that may be evaluated in this article, or claim that may be made by its manufacturer, is not guaranteed or endorsed by the publisher.
