# Supplementary material for: Nitric oxide promotes energy metabolism and protects mitochondrial DNA in peaches during cold storage
Source: Front Plant Sci. 2022 Oct 6;13:970303. doi: 10.3389/fpls.2022.970303 (PMC9582448; doi:10.3389/fpls.2022.970303)
Supplement: Supplementary file 1 [file Table_1.docx]

Supplementary Material

1. Determination of soluble sugar content, color difference, relative conductivity, weight loss, respiratory intensity and firmness in peaches.

The color difference was determined by a CR-10 colorimeter (Konica Minolta, Japan), and expressed as *ΔE*. The soluble solids content (SSC) of peaches was measured by a hand refractometer (Shanghai Cany Precision Instrument Co. Ltd, China) and expressed as °Brix. The weight was measured by electronic scales (BS124s, Sartorius, Germany). The weight loss rate was calculated with the following formula: Weight loss ratio (%) = (original weight - weight after storage) / original weight × 100. The relative conductivity was measured by conductivity meter (Lemag, China). The respiratory intensity was determined using an SY-1022 gas analyzer (Shiya Technology Co. Shijiazhuang, China), and expressed as mg kg^-1^ h^-1^. The firmness of peaches was measured by the Edelberg GY-4 fruit firmness tester (Shanghai Shandu, China) with the diameter of the probe at 8.0 mm, and expressed as N.

1.3 Results

It was found that treatment with NO at 15 μmol L^-1^ effectively inhibited the increase in color difference, soluble sugar content, weight loss, relative conductivity and respiratory intensity, and delayed the decrease in firmness in peaches (Fig. S1).





**Supplementary Figure 1.** Effects of NO treatment on color difference (A), soluble sugar content (B), weight loss (C), relative conductivity (D), respiratory intensity (E) and firmness (F) in peaches. Each value is presented as mean ± SE (n=3).
